# Docosahexaenoic Acid-Loaded Nanostructured Lipid Carriers for the Treatment of Peri-Implantitis in Rats

**DOI:** 10.3390/ijms24031872

**Published:** 2023-01-18

**Authors:** Zhen Li, Zhaoyi Yin, Baosheng Li, Jie He, Yanqun Liu, Ni Zhang, Xiaoyu Li, Qing Cai, Weiyan Meng

**Affiliations:** 1 Department of Dental Implantology, Hospital of Stomatology, Jilin University, Changchun 130021, China; 2 Jilin Provincial Key Laboratory of Oral Biomedical Engineering, Changchun 130021, China

**Keywords:** docosahexaenoic acid, inflammation, macrophage, nanostructures, peri-implantitis

## Abstract

Being the most common cause of implant failure, peri-implantitis is defined as a pathological condition associated with the occurrence of peri-implant plaque, characterized by peri-implant mucosal inflammation and progressive loss of the supporting bone tissue attributed to the persistence of pro-inflammatory cytokines. Docosahexaenoic acid (DHA), which is a type of omega-3 polyunsaturated fatty acid, is generally used for the treatment of many inflammatory diseases. However, a suitable form for dosing and its therapeutic effect on peri-implantitis remain unclear. In this study, a novel nanostructured lipid carrier (NLC) loaded with squalene and DHA was fabricated (DHA-loaded NLC). The encapsulation efficiency and drug loading efficiency values of the DHA-loaded NLC were 78.13% ± 1.85% and 28.09% ± 0.48%, respectively. The release of DHA was gradual and steady until 144 h. In addition, the free-radical-scavenging rate of DHA-loaded NLC (0.57 ± 0.03) was much higher than that of sole DHA (0.17 ± 0.003). By inhibiting nuclear factor-κB p65 nuclear translocation, DHA-loaded NLC prevented the activation of nuclear factor-κB downstream inflammatory pathways and exerted anti-inflammatory effects on macrophages. Moreover, DHA-loaded NLC showed better effects on preventing alveolar bone resorption of rat peri-implantitis model than sole DHA. Hence, DHA-loaded NLC enhanced the anti-inflammatory bioavailability of DHA, offering a novel approach for the treatment of peri-implantitis.

## 1. Introduction

Due to the popularity of implant restorations, peri-implantitis has become a major postoperative complication [1], with prevalence rates ranging from 1% to 47% [2]. Peri-implantitis is a plaque-related pathological condition that is characterized by peri-implant soft tissue inflammation and progressive alveolar bone loss [3]. The periodontal ligament of the natural tooth and the Sharpey fiber that is vertically inserted into the cementum on the surface of the natural tooth are both absent around the osseointegrated implants [4]. When plaque accumulates in large quantities due to this anatomical functional deficiency, endotoxin drives macrophages/monocytes to produce pro-inflammatory cytokines, which arouse an immunological response [5]. The large-scale production of damaging inflammatory mediators can disrupt the immune response, prolong inflammation, exacerbate tissue loss, and eventually result in implant failure [6]. Both surgical and non-surgical methods are currently being investigated for peri-implantitis treatment. Surgical removal of the bacterial biofilm is more distinct; however, it faces ineluctable risks such as painful trauma, postoperative complications, etc. [7]. Non-surgical treatments, such as mechanical and pharmacological treatments, also face disadvantages, such as incomplete removal of the bacterial biofilm, damage to the implant surface, and a tendency to develop drug-resistant strains of bacteria [8,9]. Therefore, it is a new concern to employ natural substances with minor adverse responses to limit the immune response of peri-implant tissue and decrease tissue damage.

Omega-3 polyunsaturated fatty acid (PUFA), an essential PUFA found in large amounts in cod liver oil and seaweed [10], is widely used to treat inflammatory diseases, such as cardiovascular diseases [11], inflammatory bowel diseases [12], and the inflammation caused by obesity [13]. A systematic review showed that dietary supplementation with omega-3 fatty acids as a supplement to nonsurgical periodontal therapy (SRP) in patients with periodontitis was more effective than SRP alone in reducing periodontal disease and improving the clinical attachment level [14]. The main members of the omega-3 family are alpha-linolenic acid, eicosapentaenoic acid (EPA), and docosahexaenoic acid (DHA). It has been suggested that DHA is superior to EPA for lowering the production of cytokines by activated macrophages in vitro [15]. In another study, DHA inhibited osteoclast differentiation induced by RANKL better than EPA [16]. Therefore, it is reasonable to assume that DHA may exert a positive therapeutic effect in response to rapidly progressing peri-implantitis. However, the highly unsaturated structure of DHA makes it extremely susceptible to oxidation, resulting in odor alterations and loss of biological activity [17]. Based on this, an antioxidant could be used to act synergistically with DHA. Squalene, a naturally occurring biocompatible triterpenoid, is found in the liver oil of several fish species, particularly deep-sea sharks. The antioxidant effects of squalene have been demonstrated in vitro and in vivo by reducing stress-induced intracellular reactive oxygen species (ROS), cytokine release, and the global expression of pro-inflammatory genes in immune cells [18,19]. Based on its excellent biological activity, squalene is frequently used as an adjuvant in vaccines to boost the immune system’s response to antigens and as a lipid matrix in the production of nanomedicine [20]. Nevertheless, DHA and squalene are both water-insoluble oily liquids. As a consequence, they cannot be readily united. 

Nano drug-delivery systems, such as nanoemulsions, are effective ways to protect, transport, and address bioactivity and insolubility [21]. A novel type of drug-delivery system called a nanostructured lipid carrier (NLC) was created on the foundation of solid lipid nanoparticles (SLNs). NLCs offer favorable mass production, high stability, better biocompatibility, higher drug loading, and a higher encapsulation rate while reducing drug leakage and abrupt release due to lipid crystallization [22]. Therefore, NLCs carrying squalene and DHA may be suitable for the treatment of peri-implantitis.

**Figure 1:**
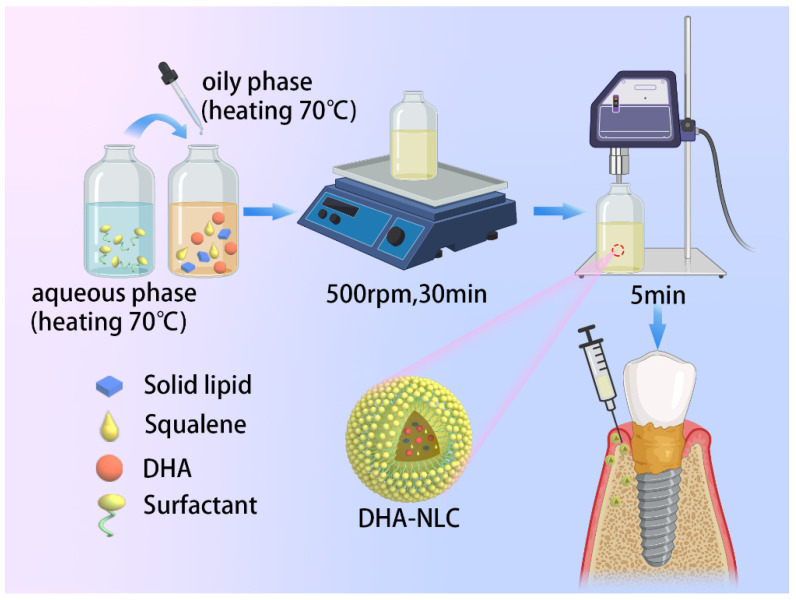
Schematic diagram of the preparation and application of the DHA-NLC. A DHA-loaded NLC was fabricated using heating and ultrasonication methods. A surfactant constituted the peripheral spherical shell, while DHA and squalene were encapsulated inside. DHA-loaded NLC exerted anti-inflammatory effects in a rat model of peri-implantitis by attenuating the pro-inflammatory factor levels and preventing alveolar bone resorption. NLC, nanostructured lipid carrier. DHA, docosahexaenoic acid.

In this study, squalene was used as liquid lipid, and the required DHA-loaded NLC was prepared by the phacoemulsification method to determine its anti-inflammatory effect and mechanism in LPS-stimulated macrophage inflammation and rat peri-implant inflammation models (Figure 1). The null hypothesis is that the DHA-loaded NLC prepared in the experiment has no anti-inflammatory effect. This study is expected to provide guidance for the treatment of clinical peri-implantitis.

## 2. Results

### 2.1. DHA-Loaded NLC Was Fabricated, Which Had Uniform Particles, Slow Release, and ROS Resistance 

**Figure 2:**
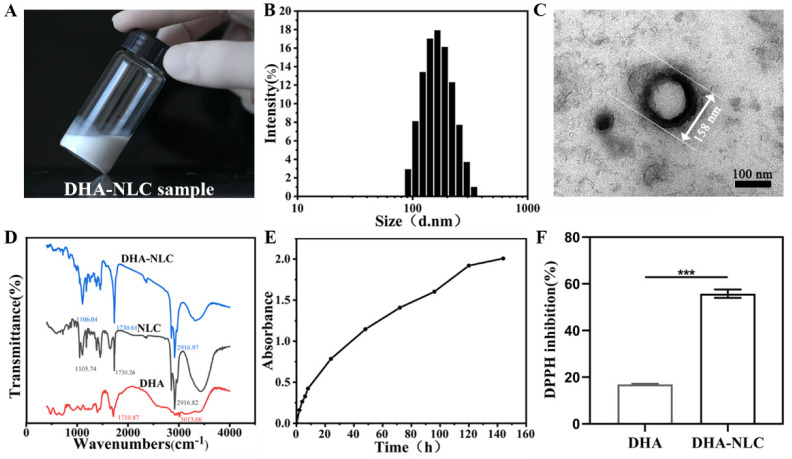
Photographic image and characterization of DHA-NLC. (**A**) Emulsibility of DHA-NLC. (**B**) Particle sizes of DHA-NLC. (**C**) TEM of DHA-NLC. Scale bar: 100 nm. (**D**) FITR spectra of NLC, DHA, and DHA-NLC. (**E**) Release curve of DHA-NLC. (**F**) DPPH inhibition rate of DHA and DHA-NLC. LPS, lipopolysaccharide. *** *p* < 0.001. NLC, nanostructured lipid carrier. DHA, docosahexaenoic acid.

In this study, a homogeneous nanoemulsion (Figure 2A) with average dimensions of 163.7 ± 2.0 nm, a PDI of 0.118 ± 0.01, and zeta potential of 40.1 ± 1.3 mV was created (Figure 2B). The representative morphology of the DHA-loaded NLC is shown in Figure 2C, represented as a spherical particle with a 158 nm diameter. Moreover, there was a spherical shell with high density, while a low-density substance could be seen internally. The DHA-loaded NLC encapsulation efficiency (EE) and drug loading efficiency (LC) values were 78.13% ± 1.85% and 28.09% ± 0.48%, respectively. When the DHA-loaded NLC was kept at 4 °C for a month, neither precipitation nor crystallization were seen. Over a month, there were no appreciable changes in the zeta potential or particle size of the DHA-loaded NLC.

The frequency regions evident on the DHA spectra were compared between the DHA-loaded NLC and NLC with the help of FTIR spectra. The characteristic peaks of DHA were the stretching vibration peaks of the C=O of fatty acid lipid bonds at 1712 cm^−1^ and C=H at 3013 cm^−1^ (Figure 2D). It can be seen from the infrared spectrum of the DHA-loaded NLC that the characteristic peak of DHA shifted or disappeared, indicating that the stretching vibration or bending vibration of the group after the DHA was encapsulated was limited. Furthermore, the infrared spectra of the DHA-loaded NLC and the NLC showed the same characteristic peaks, with no additional absorption peaks. Therefore, the DHA was completely encapsulated in the NLC.

During the initial 8 h in terms of drug release, DHA was released almost linearly from the DHA-loaded NLC. Following this, the release of DHA became gradually stable until 144 h (Figure 2E).

DPPH showed that the free radical scavenging rate was 0.17 ± 0.003 for the DHA and 0.57 ± 0.03 for the DHA-loaded NLC (Figure 2F).

### 2.2. DHA-Loaded NLC Exerted Anti-Inflammatory Effects on Macrophages through NF-κB Pathway In Vitro

#### 2.2.1. Cytotoxicity Assay

**Figure 3:**
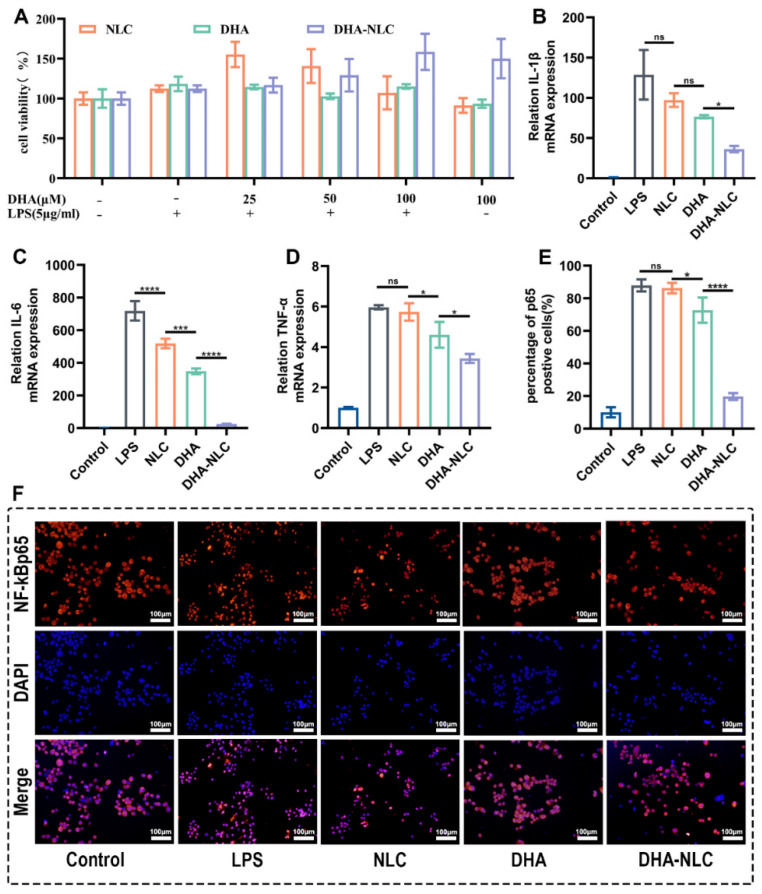
Cytotoxicity and anti-inflammatory effects of each group in vitro. (**A**) Viability of RAW 264.7 cells cultured with NLC, DHA, and DHA-NLC at different concentrations. (**B**–**D**) Effects of each group on the mRNA expression of *IL-1β*, IL-6, and *TNF-α* in macrophages in vitro (n = 3). (**E**,**F**) NF-κB p65 translocation in macrophages was detected by immunofluorescence, and positive cells were counted (n = 3). * *p* < 0.05, *** *p* < 0.001, **** *p* < 0.0001. ns: no statistical significance. Scale bars: 100 μm. LPS, lipopolysaccharide. NLC, nanostructured lipid carrier. DHA, docosahexaenoic acid.

According to the Cell Counting Kit-8 data (Figure 3A), the NLC, DHA, and DHA-loaded NLC had no significant impact on macrophage viability at the concentrations examined, indicating that the anti-inflammatory effects exerted by these materials were not the result of their non-specific cytotoxic effects.

#### 2.2.2. Effect of DHA-Loaded NLC on the Expression of Cellular Inflammatory Factors

Compared with the control group, macrophages that were challenged by *P. gingivalis* LPS displayed a significant inflammatory state. NLC showed no significant effect on inhibiting the expression of *IL-1β*, and *TNF-α*. DHA-loaded NLC showed a better inhibitory effect than sole DHA on *IL-1β*. Meanwhile, the NLC group showed only a slight inhibitory effect compared with the DHA group (Figure 3B). Similarly, for *IL-6* and *TNF-α*, the DHA-loaded NLC displayed stronger anti-inflammatory effects (Figure 3C,D). 

#### 2.2.3. Effects of DHA-Loaded NLC on LPS-induced NF-κB Activation

It was clear that the nuclear translocation and positive expression of p65 occurred in the LPS and NLC groups of macrophages as opposed to healthy macrophages, proving that LPS was successful in activating the NF-κB signaling pathway. Additionally, compared with the DHA group, the DHA-loaded NLC group showed a more pronounced suppression of NF-κB p65 nuclear translocation (Figure 3E,F). This is in line with the result that DHA-loaded NLC had stronger anti-inflammatory benefits.

### 2.3. DHA-Loaded NLC Exerted Anti-Inflammatory Effects on Rat Peri-Implantitis In Vivo

**Figure 4:**
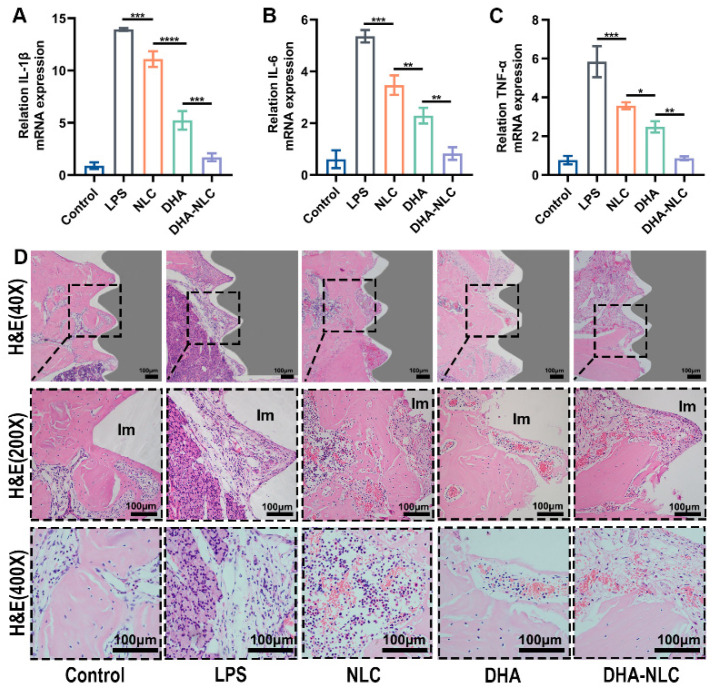
Anti-inflammatory effects of each group in peri-implantitis in vivo. (**A**–**C**) Effects of each group on the mRNA expression of *IL-1β*, *IL-6*, and *TNF-α* in peri-implant tissue. The results are presented as the mean ± SD (n = 3). (**D**) Representative H&E staining images of peri-implant tissues from each group (grey parts represent the implants). The dashed line box in the HE (40×) image is the selected viewing area, and the HE (200×) and HE (400×) are enlargements of this section. * *p* < 0.05, ** *p* < 0.01, *** *p* < 0.001, **** *p* < 0.0001. Scale bars: 100 μm. LPS, lipopolysaccharide. NLC, nanostructured lipid carrier. DHA, docosahexaenoic acid. Im, implant.

The qRT-PCR results showed elevated expression of inflammatory factors *IL-1β*, *IL-6*, and *TNF-α* in the gingival tissue of the inflammatory group compared with the control group (Figure 4A–C). This is in line with the soft tissue swelling, redness, and bleeding that were observed during the aforementioned modeling procedure. After 2 weeks of reagent treatment, the rats in the NLC and DHA groups had lower levels of gingival inflammatory factors than those in the LPS group. Meanwhile, the DHA group showed slightly better effects than the NLC group. Further, the DHA-NLC group showed the best effects on repressing inflammation. The concentrations of *IL-1β*, *IL-6*, and *TNF-α* in the DHA-NLC group were nearly identical to those in the control group.

Hematoxylin–eosin (H&E) staining proved that there was almost no inflammatory tissue in the control group. In contrast, peri-implantitis lesions in the LPS group showed the most pronounced inflammation, manifested as higher densities, numbers, and area proportions of plasma cells, macrophages, as well as neutrophils. Meanwhile, inflammatory cell infiltration throw was relatively obvious in the NLC group, while the DHA and DHA-NLC groups showed a significant reduction and basic disappearance of inflammation in the peri-implant tissues. (Figure 4D).

### 2.4. DHA-Loaded NLC Exerted Osteoprotective Effects on Rat Peri-Implantitis In Vivo

**Figure 5:**
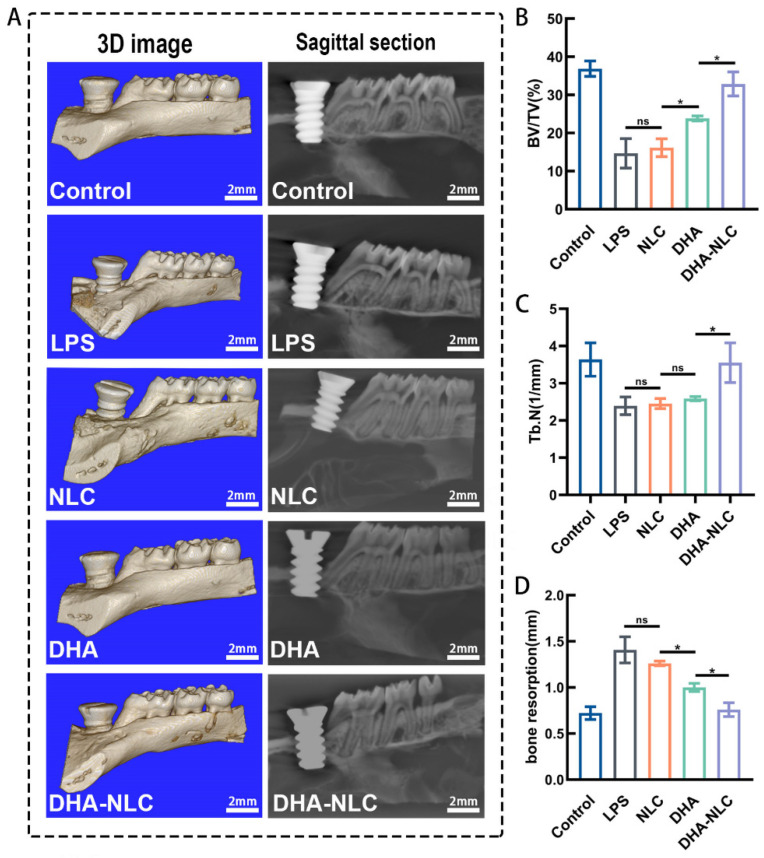
Effect of each group on bone resorption in the peri-implantitis model in vivo. (**A**) Micro-CT images of the rat alveolar bone. (**B**,**C**) Bone morphometric parameters: bone volume/tissue volume (BV/TV) and trabecular number (Tb.N) around the titanium implants (n = 3). (**D**) Bone resorption level (n = 3). Scale bars: 2 mm. * *p* < 0.05. LPS, lipopolysaccharide. NLC, nanostructured lipid carrier. DHA, docosahexaenoic acid.

The micro-CT results are shown in Figure 5. The image with a blue background is the 3D reconstruction image. The image with a black background is a representative sagittal micro-CT section (Figure 5A). All implants achieved stable osseointegration at the anterior side of the maxillary left first molar. In the control group, the implant had good osseointegration with the alveolar bone. The distance between the implant shoulder and bone-to-implant contact was defined as the DIB, which was considered as the bone resorption in this study. The region of interest was chosen as a cylinder with a 2 mm diameter and 2.2 mm height, with the implant’s center acting as the axis. Compared with the control group, the LPS group showed a 57.71% decrease in the bone volume/tissue volume (BV/TV) ratio and 94.92% increase in bone resorption. Meanwhile, the NLC group did not show a significant difference with the LPS group (Figure 5B–D). The BV/TV and the bone trabeculae (Tb.N) of the NLC, DHA, and DHA-NLC groups showed a sequentially increasing tendency, and the bone absorption values correspondingly followed the opposite tendency (Figure 5B,D). In particular, the BV/TV, Tb.N, and bone resorption of the DHA-NLC group did not show any significant differences with the control group (Figure 5B–D).

### 2.5. DHA-Loaded NLCs Represented Good Biocompatibility In Vivo

**Figure 6:**
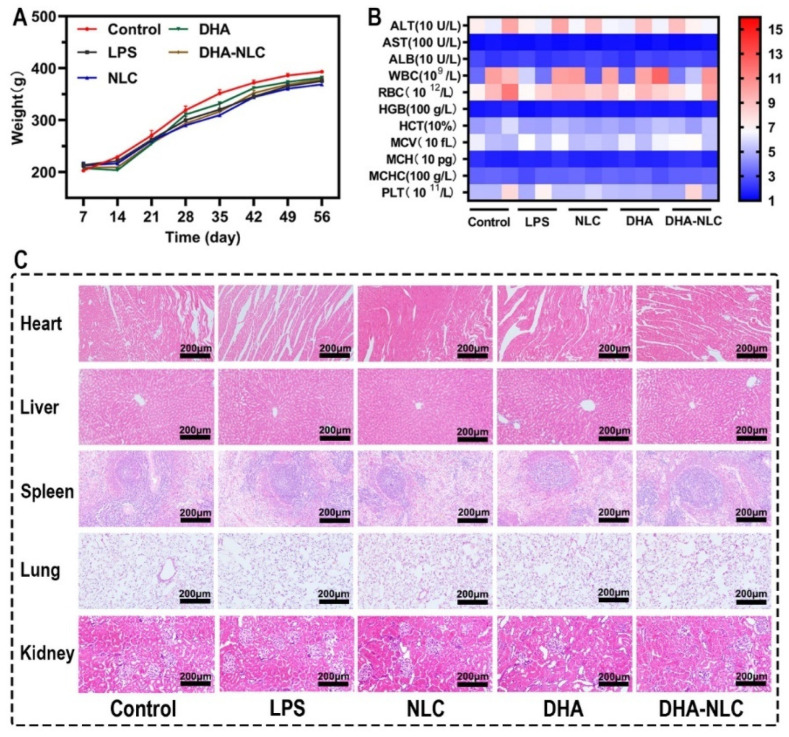
Biosafety of each group in vivo. (**A**) Effect of each group on rat weight during the experimental period. (**B**) Effect of each group on alanine aminotransferase (ALT), aspartate aminotransferase (AST), albumin (ALB), white blood cells (WBC), red blood cells (RBC), hemoglobin (HGB), hematocrit (HCT), mean corpuscular volume (MCV), mean corpuscular hemoglobin (MCH), mean corpuscular hemoglobin concentration (MCHC), and platelet (PLT) levels. (**C**) H&E staining of the heart, liver, spleen, lung, and kidney tissues. Scale bars: 200 μm. LPS, lipopolysaccharide. NLC, nanostructured lipid carrier. DHA, docosahexaenoic acid.

The rats in the experimental group continuously increased in weight throughout the experiment, except for the initial stages of implantation and ligation (Figure 6A). In addition, no significant differences were observed in the results of routine blood and liver function tests, and H&E staining of the rats’ organs (Figure 6B,C).

## 3. Discussion

In order to validate the role of DHA in peri-implantitis and to improve its bioavailability, a nanostructured lipid carrier with DHA encapsulated using squalene as a liquid lipid was developed in this study. The common preparation methods of NLC include high-pressure homogenization, microemulsion, the solvent injection method, phase inversion, and ultrasonic emulsification [23]. The ultrasonic emulsification method was chosen in this experiment for its simplicity, lack of large experimental equipment, and lack of organic solvent residue. The average particle size of the DHA-loaded NLCs obtained in our experiment was 167 ± 2 nm, with a uniform shape and smooth surface, and no obvious particle aggregation. The encapsulation rate (78.13 ± 1.85%) and drug loading rate (28.09 ± 0.48%) were satisfactory. The PDI value (0.118 ± 0.01) was less than 0.3, indicating a narrow particle size distribution and good dispersion homogeneity, which was in agreement with the transmission electron microscopy (Figure 5). The zeta potential value (40.1 ± 1.3 mV) of the NLCs after DHA loading still exceeded 30 mV, indicating that there was sufficient electrostatic repulsion between the DHA-loaded NLC particles to maintain the stability of the whole system [24]. Long-term stability studies of the drug showed that DHA-loaded NLC could remain stable for at least one month, which may be due to the use of Tween 80 and glycerol surfactant during preparation. According to the DPPH results, the antioxidant effect of DHA-loaded NLC was attributed to the encapsulated structure of nanostructured lipid carriers and the incorporation of squalene compared with DHA.

In this experiment, a series of studies was carried out to verify the effect of DHA-loaded NLC on peri-implantitis in vitro and in vivo. RAW 264.7 macrophages dramatically increased their expression of inflammatory factors when exposed to *P. gingivalis* LPS at 5 μg/mL. The pro-inflammatory response was suppressed to varying degrees in each experimental group when the dose was 50 μg/mL, according to the qRT-PCR data. Additionally, this concentration was inside the drug’s acceptable limit. In comparison to the DHA and NLC groups, DHA-loaded NLC had a larger impact on the decreases in *IL-1β*, *IL-6*, and *TNF-α*. Previous animal studies demonstrated that wrapping a ligature around the implant neck in a submucosal position accelerated plaque formation, leading to the development of peri-implantitis lesions, similar to the development of natural diseases [25]. In another study, peri-implantitis was established by injecting LPS, an endotoxin produced by peri-implantitis-associated bacteria, around the implant to induce a rapid and stressful immune response in the host [26]. The present study combined the advantages of both of the above approaches, confirming the success of the constructed rat peri-implantitis model. In comparison with DHA alone and NLC, DHA-loaded NLC had a better therapeutic effect on rat peri-implantitis. Additionally, the DHA-loaded NLC group was able to reduce alveolar bone resorption, which is consistent with the changes in inflammatory mediators, according to the micro-CT results. This may have been due to the slow release of DHA, resulting in a sustained suppression of inflammation in the local microenvironment. The timely resolution of inflammation, in turn, led to bone reconstruction at the implant interface [27]. Therefore, based on the above experimental results, the null hypothesis is invalid.

Continuous inflammation, which results from an imbalance in the immune system’s control, is key to the degeneration of the peri-implant tissue. The development and progression of peri-implantitis are significantly influenced by different immune cells, as well as the precise expression of different immune cells and cytokines [28]. As a typical pro-inflammatory cytokine, IL-1β is involved in immune regulation, affects connective tissue metabolism, causes inflammation, stimulates the creation and maturation of osteoclasts, and inhibits bone formation in peri-implantitis by triggering osteolysis [29]. Il-1β is also involved in controlling the production of prostaglandin E2 to affect bone resorption [30]. The early release and quick synthesis of TNF-α recruit other cells to the inflammation site and directly contribute to bone resorption by increasing osteoclast precursors in the bone marrow [31]. IL-6 increases osteoblast-mediated osteoclast differentiation through the activation of JAK2 and RANKL, thereby accelerating bone resorption [32]. However, DHA treatment can reverse them. As a type of unsaturated fatty acid, DHA can inhibit many aspects of inflammation, such as leukocyte chemotaxis, adhesion molecule expression, and leukocyte–endothelial adhesion interactions. They can also alter the composition of lipid fatty acids in cell membranes, disrupt lipid rafts, and produce some pro-inflammatory mediators of resolution [33,34]. In terms of the signaling pathways that control gene expression in inflammatory cells, the NF-κB family plays a key role in the regulation of both innate and acquired immunity. In normal resting cells, NF-κB typically exists as a heterodimer in the cytoplasm. The complex, which is made up of the p50 and p65 subunits, binds to a class of repressor proteins known as inhibitor kappaB (IκB). Upon stimulation, IκB is degraded, the P65 subunit of NF-κB is transported from the cytosol to the nucleus, and NF-κB is activated. NF-κB activation triggers a signaling cascade in which many chemokines, cytokines, adhesion molecules, inflammatory mediators, and apoptosis suppressor genes are expressed [35,36]. According to the immunofluorescence findings, DHA-loaded NLC better exhibited a definite anti-inflammatory function and suppressed NF-κB P65 nuclear translocation compared with other groups. This is in line with Won-Gun Kwack’s research, which demonstrated that simultaneous aspirin pretreatment and enteral supplementation with omega-3 fatty acids reduced lung injury in a VILI mouse model by reducing NF-κB activation [37].

The nanostructured lipid carrier in this experiment was considered to be the reason for the superior effect of the DHA-loaded NLC group compared with DHA alone. Meanwhile, the minimal anti-inflammatory and osteoprotective effects of the NLC group were attributed to the liquid lipid squalene in the nanostructured lipid carrier. The creation of multifunctional nanoparticles with anti-inflammatory properties using the natural lipid squalene and the endogenous immunomodulator adenosine managed endotoxemia in rodents [38]. This is consistent with the role played by squalene in this experiment. The oral cavity is an open and aerobic environment, and the treatment of oral diseases is also unique. Because of their resistance to environmental impacts (oxygen, light, humidity, gastric acid, etc.), DHA is currently frequently utilized clinically as an oral dietary supplement in the form of fish oil capsules [39]. However, the content of DHA in commercially available fish oil capsules is quite low. After oral administration, DHA must be transported through the systemic circulation to the periodontal tissue, and it is also susceptible to gastrointestinal metabolism and inactivation during this process [40]. Therefore, it is difficult to determine the exact time and local concentrations of DHA that enter the periodontal tissues. The large doses of the drug required to achieve the minimum effective concentration may lead to adverse effects, such as toxicity, gastrointestinal intolerance, and drug resistance. In contrast, the DHA-loaded NLC in this study not only combined the advantages of squalene and DHA with higher bioavailability and biosafety, but also improved drug loading. Moreover, the structural characteristics of lipid nanoparticles enable them to preferably penetrate cell membranes and facilitate the entry of more compounds into cells [41]. Likewise, the small particles of DHA-loaded NLCs also provided a convenient way to enter cells to perform their functions. Further, DHA-loaded NLCs also have a large number of tiny liquid oil compartments dispersed throughout the solid matrix [42]. The compartments were surrounded by a solid lipid matrix, which further delayed the release. 

Comprehensively, this study innovatively prepared DHA-loaded NLCs by compounding squalene as an antioxidant with DHA, giving full play to its structural advantages, enabling slow local release and providing high concentrations of the drug to exert anti-inflammatory effects with fewer side effects and higher safety. This innovative form of DHA overcame the drawbacks of DHA, including quick oxidation and insolubility in water. Additionally, DHA-loaded NLCs may transform the administration form of DHA from oral medication to local administration, opening a new avenue for the treatment of peri-implantitis and providing a new technique for overcoming complex and multifactorial inflammatory phenomena.

Nonetheless, there are some limitations of this experiment based on the aforementioned studies. For instance, DHA-loaded NLCs were injected into the inflammatory gingival tissue; therefore, the injection dose was extremely limited. Moreover, this manner of administration would cause some discomfort and even pain, which needs further optimization and improvement of the dosage form. In addition, large animals, such as domestic pigs, should be furtherly employed in animal experiments instead of rodents to better inform clinical practice. 

## 4. Materials and Methods

### 4.1. DHA Nanoencapsulation and Characterization

#### 4.1.1. DHA-Loaded NLC Preparation

DHA-loaded NLCs were fabricated through ultrasonication using 800 mg of monostearin (Yuanye Biotechnology Co., Shanghai, China), 200 mg of squalene (Yuanye Biotechnology Co., Shanghai, China), 500 mg of DHA (Maclean Biochemical Technology Co., Shanghai, China), 270 mg of Tween 80 (Sinopharm Group Chemical Reagent Co., Ningbo, China), 30 mg of glycerol (Xintong Fine Chemical Co., Tianjin, China), and 7200 mg of ultrapure water. Briefly, ultra-pure water was added to an oil phase composed of two lipids (monostearic acid and squalene), DHA, and two surfactants (Tween 80 and glycerol). Then, the reagents were heated together at 70 °C to promote binding. Subsequently, the liquid was agitated for 30 min at 500 rpm (IKA Instruments & Equipment Co., Guangzhou, China) to create a milky pre-emulsion. The DHA-loaded NLCs (DHA-NLC) were then obtained by using a sonicator (Scientz Biotechnology Co., Ningbo, China) at 60% amplitude for 5 min.

#### 4.1.2. Transmission Electron Microscopy Analysis

The morphology of the DHA-loaded NLCs was observed using transmission electron microscopy (FEI-Tecnal spirit, Hillsboro, OR, USA). The sample was obtained by dropping a small amount of the formulation onto a 100-mesh copper mesh and then diluting it 50 times with 3% phosphotungstic acid for negative staining.

#### 4.1.3. DHA-Loaded NLC Size and Charge Measurement

A Zetasizer Nano-ZS90 was used to measure the particle size, polydispersity index (PDI), and zeta potential of the DHA-loaded NLCs (Malvern, Egham, Britain). The test samples were diluted 5 times with deionized water at 25 °C. All samples were tested three times.

#### 4.1.4. Determination of Encapsulation Efficiency (EE) and Drug Loading Efficiency (LC)

UV/VIS-spectroscopy (Shimadzu Instruments Co., Suzhou, China) was used to quantify the DHA nanoencapsulation. DHA-loaded NLCs were dissolved in n-hexane for 30 min, centrifuged for 30 min at 12,000 rpm, and then evaluated by measuring the absorbance of the supernatant at 272 nm using a UV/VIS spectrophotometer. By determining the amount of free DHA in the DHA-loaded NLC dispersions using the following equations, EE and LC were indirectly calculated:

EE (%) = (Total DHA − free DHA)/Total DHA × 100%

LC (%) = (Total DHA − free DHA)/(free DHA + Total lipid) × 100%

#### 4.1.5. FTIR Analysis

Using an FTIR spectrometer (Thermo Scientific, Waltham, USA) with a resolution of 4 cm^−1^ in 64 scans, FTIR spectroscopy of the DHA, blank-NLCs, and DHA-loaded NLCs was conducted. The sample was combined with potassium bromide powder (Guangfu Fine Chemical Research Institute, Tianjin, China) and a thin plate of potassium bromide served as the background. A pellet of the combination was formed and measured by FTIR in the 400–4000 cm^−1^ range.

#### 4.1.6. Free Radical Scavenging Experiments with 2,2-diphenyl-1-propenyl Hydrazide 

With a few minor alterations, this experiment was based on Qayyum Shehzad’s methodology [21]. The ability of the DHA and DHA-loaded NLCs to scavenge free radicals was tested using the 2,2-diphenyl-1-propenyl hydrazide (DPPH) assay. In anhydrous ethanol, a 200 M DPPH (Maclean Biochemical Technology Co., Shanghai, China) solution was prepared. A 3.5 mL DPPH solution was combined with approximately 0.5 mL of DHA-loaded NLCs or DHA–anhydrous ethanol solution. Ten min of darkness and room temperature were required for the test. The following equations were calculated by UV/VIS spectroscopy:

%DPPH inhibition = [(ABS_Blank_ − ABS_Sample_)/ABS_Blank_] × 100

#### 4.1.7. In Vitro Drug Release

The release profile of DHA from the DHA-loaded NLCs was assessed using a dynamic dialysis technique. Then, 2 mL of DHA-loaded NLCs was added to a dialysis bag. The dialysis bag was then placed in a centrifuge tube with 40 mL of PBS solution and 1% Tween 80 as the dialysis medium. In a thermostatic gas bath, the centrifuge tube was maintained at 37 °C and 150 rpm. One milliliter of the release solution was swapped out for the same amount of dialysis medium at predefined intervals. A UV/VIS spectrophotometer was used to assess the absorption at 276 nm.

#### 4.1.8. DHA-Loaded NLC Stability

The drug was stored in a refrigerator at 4 °C and the surface charge and dimensions were periodically measured to assess its stability.

### 4.2. Cytotoxicity Assay

Macrophages were provided by the Shanghai Institute of Cellular Sciences, China. Briefly, macrophages were cultured in Dulbecco’s Modified Eagle Medium (HyClone, Logan, UT, USA) with 10% fetal bovine serum (Biological Industries, Haemek, Israel) and 1% penicillin–streptomycin solution at 37 °C with 5% CO_2_ in a humidified cell incubator. Cultured cells that were 70% confluent were used. Afterward, 96-well plates were inoculated with 1 × 10^4^ macrophages per well and cultured for 12 h. Following 2 h of pretreatment with each drug group, the cells were stimulated with *P. gingivalis* LPS (InvivoGen San Diego, CA, USA) for 24 h. (The drug concentration was based on the concentration of DHA in the DHA-loaded NLCs. The concentrations of the DHA solution and NLC should be consistent with those of DHA-loaded NLC.) Cell Counting Kit-8 (NCM Biotech, Suzhou, China) solution (10 μL) was added to the wells and incubated for 2 h at 37 °C. A microplate reader (Bole Life Medical Products, Shanghai, China) was used to determine the absorbance at 450 nm.

### 4.3. Quantitative Real-Time Polymerase Chain Reaction In Vitro

The procedure for macrophage cultivation was the same as that mentioned above. The cell inoculation density was 2 × 10^5^ cells/well, and 50 μM was set as the final concentration in the formal experiment by screening in the pre-experiment. Trizol reagent (YEASEN, Shanghai, China) was used to extract the total RNA, and Hifair^®^ III 1st Strand cDNA Synthesis SuperMix for quantitative polymerase chain reaction (qPCR) (gDNA Digester+) (YEASEN, Shanghai, China) was used to synthesize cDNA for the Roche LightCycler^®^ 96 RT-qPCR platform’s real-time polymerase chain reaction (PCR) (Roche, Basel, Switzerland). The primer sequences used for qRT-PCR are listed in Table 1, with mouse *TNF-α*, *IL-1β*, and *IL-6* as the specific primers and *β-actin* mRNA as an internal reference.

### 4.4. Immunofluorescence

As previously mentioned, the macrophages were cultured. Drugs were co-cultured with 1.5 × 10^4^ cells per well for 24 h. Then, the macrophages were stimulated with LPS (5 μg/mL) for 3 h. The cells were collected, fixed, and permeabilized sequentially. Thereafter, the cells were incubated with nuclear factor-κB (NF-κB) p65 (1:100, Abcam) overnight at 4 °C and then rabbit Alexa Fluor 455 anti-mouse antibody (1:1000, Beyotime) for 1 h on the following day at room temperature. The number of NF-κB p65-positive cells was observed and measured under an Olympus microscope.

### 4.5. Animal Experiment Arrangement

Male adult Wistar rats (n = 60, weight: 200–220 g) were kept at room temperature (22 °C ± 2 °C) with a 12:12 light/dark cycle and had free access to food and water. The First Hospital of Jilin University’s Animal Ethics Committee approved the use of animals in this experiment (No. 2022-0576).

### 4.6. Establishment of Peri-Implantitis Model 

After sequential cleaning, surface sandblasting, large-grit etching, and acid etching (SLA), titanium SLA mini-implants (diameter: 1.8 mm, length: 3.7 mm) were obtained (WEGO, Weihai, China) (Supplementary Figure S1). The rats were subjected to general anesthesia with 2% sodium pentobarbital. Then, the implants were placed on the anterior side of the maxillary left first molar after elevating the full-thickness flap. The rats were administered benzylpenicillin sodium (80,000 units/1 rat) for 3 days to avoid postoperative infection. The rats were randomly divided into 5 groups (n = 12) 1 month after implant placement: healthy rats (control group), rats with peri-implantitis (LPS group), rats with peri-implantitis treated with NLCs (NLC group), rats with peri-implantitis treated with DHA (DHA group), and rats with peri-implantitis treated with DHA-loaded NLCs (DHA-NLC group). The implant neck was then ligated with 4-0 silk, and 10 µL of 1 mg/mL *P. gingivalis* LPS was injected into the peri-implant fissure every 3 days in the following 2 weeks to establish peri-implantitis. The peri-implant gingival tissue was then immediately injected with 100 μL of each group emulsion using an insulin syringe (KDL, Shanghai, China) at the four points of the implant (mesial, distal, buccal, and palatal) every 3 days within 2 weeks. Afterward, 4% paraformaldehyde was used to fix the dissected rat maxillae before performing microcomputed tomography (micro-CT) and hematoxylin and eosin (H&E) staining. For qRT-PCR, the peri-implant gingival tissue was extracted. The centrifuged serum and right ventricle blood samples were used to evaluate liver function and perform regular blood tests. Hearts, livers, spleens, and kidneys were also harvested for in vivo toxicity testing.

### 4.7. Micro-CT

Micro-CT (Scanco CT50, Bassersdorf, Switzerland) was used to analyze the bone tissue. The scanning parameters were as follows: voxel size of 34.4 μm, 200 mA, 70 kVp, and exposure time of 300 ms. The trabecular thickness (Tb. Th, mm) and bone volume fraction (bone volume/tissue volume, BV/TV, %) within the regions of interest were analyzed.

### 4.8. qRT-PCR In Vivo

Magnetic beads were used to grind the peri-implant gingival tissue, and TRIeasy was used to extract the mRNA. The synthesis of cDNA and qRT-PCR was performed referring to the methods mentioned above. To compare the relative mRNA expression levels, the in vivo qRT-PCR primers are listed in Table 2.

### 4.9. Histopathological Study

After rinsing under flowing water and fixing with paraformaldehyde, the maxillae were decalcified with a 10% EDTA solution at 4 °C for 2 months. The EDTA solution was refreshed every 3 days. The implants were removed using an implanted spanner once the decalcification process was finished. Then, 2 μm tissue sections were stained with H&E and the amount of inflammatory cell infiltration was measured to determine the degree of inflammation in the gingival tissue surrounding the implants.

### 4.10. Biosecurity In Vivo

Blood tests, albumin, alanine aminotransferase, and aspartate aminotransferase analyses were performed. Following fixation, histopathological analysis was used to evaluate the drug toxicity in the primary organs of rats.

### 4.11. Statistical Analysis

All experimental results were statistically analyzed using Prism 8.0 (GraphPad, San Diego, CA, USA), and they were presented as the mean ± standard deviation (SD). One-way analysis of variance was used to evaluate the differences between groups, and a *p*-value of <0.05 was considered statistically significant. There were three iterations of each experiment in this study.

## 5. Conclusions

DHA-loaded NLCs overcome the drawbacks of DHA, including quick oxidation and insolubility in water. Additionally, DHA-loaded NLCs represent a unique approach to intercept the pathogenicity of oxidative stress to inflammation through suppressing the NF-κB pathway. Local delivery of DHA-loaded NLCs provides a novel strategy for the application of DHA for the treatment of peri-implantitis, as well as other oxidative stress-related inflammatory diseases, in clinical practice. 

## Figures and Tables

**Table 1:** Primer sequences for qRT-PCR in vitro.

Gene	Forward/Reverse Primer
*IL-1β*	F:GCCACCTTTTGACAGTGATGAG R:AGCTTCTCCACAGCCACAAT
*IL-6*	F:TCCAGTTGCCTTCTTGGGAC R:GTACTCCAGAAGACCAGAGG
*TNF-α*	F:CGTTCGTAGCAAACCACCAAG R:TTGAAGAGAACCTGGGAGTAGACA
*β-actin*	F:GGAGATTACTGCCCTGGCTCCTA R:GACTCATCGTACTCCTGCTTGCTG

**Table 2:** Primer sequences for qRT-PCR in vivo.

Gene	Forward/Reverse Primer
*Rat IL-1β*	F:CAGGATGAGGACCCAAGCAC R: GTCGTCATCATCCCACGAGT
*Rat IL-6*	F: CCGGAGAGGAGACTTCACAG R: CAGAATTGCCATTGCACAAC
*Rat TNF-α*	F: CATGATCCGAGATGTGGAACTGGC R: CTGGCTCAGCCACTCCAGC
*Rat β-actin*	F:GAGAGGGAAATCGTGCGTGAC R: ACAGGTGGAAGGTCGTCTAC

## Data Availability

Data available on request due to restrictions, e.g., privacy or ethical. The data presented in this study are available on request from the corresponding author.

## Supplementary Materials

The following supporting information can be downloaded at: https://www.mdpi.com/article/10.3390/ijms24031872/s1.
