# Supplementary figures and images for: Docosahexaenoic Acid-Loaded Nanostructured Lipid Carriers for the Treatment of Peri-Implantitis in Rats
Source: Int J Mol Sci. 2023 Jan 18;24(3):1872. doi: 10.3390/ijms24031872 (PMC9915434; doi:10.3390/ijms24031872)

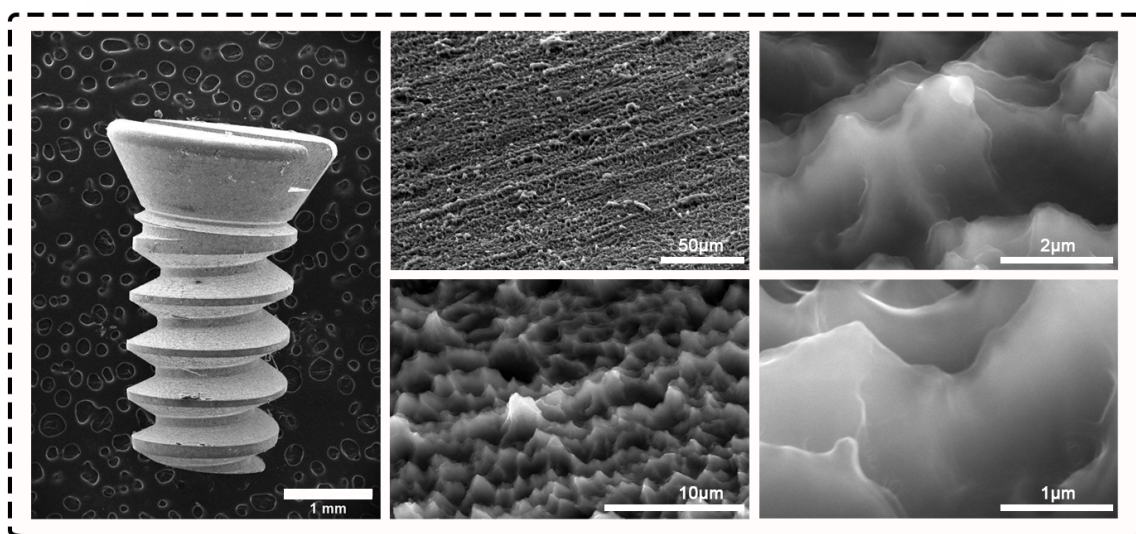

**Figure S1.** Morphological characteristics of the mini-implant.

Supplement: Supplementary file 1 [file ijms-24-01872-s001.zip › ijms-2049296-supplementary.pdf]
